# Integration of AI-driven ultrasound and molecular testing to improve diagnosis of indeterminate thyroid nodules

**DOI:** 10.3389/fendo.2026.1764495

**Published:** 2026-09-25

**Authors:** Samantha Cortes, Cameron Hrabak, Arman Danesh, Karen Jiang, Adarsh Mahendrakumar, Precious Ochuwa Imokhai, Hafiah Eltahir

**Affiliations:** 1 Florida International University Herbert Wertheim College of Medicine, Miami, FL, United States; 2 Midwestern University College of Health Sciences, Glendale, AZ, United States; 3 Arizona College of Osteopathic Medicine, Midwestern University, Glendale, AZ, United States; 4 University of California, Irvine, Irvine, CA, United States; 5 Rocky Vista University, Parker, CO, United States; 6 Michigan State University College of Osteopathic Medicine, East Lansing, MI, United States; 7 Rocky Vista University Montana College of Osteopathic Medicine, Billings, MT, United States; 8 North Atlanta Ear, Nose, and Throat and Allergy, Atlanta, GA, United States

**Keywords:** artificial intelligence, Bethesda III/IV, indeterminate cytology, radiomics, risk stratification, thyroid nodules

## Abstract

**Background:**

Indeterminate thyroid nodules (Bethesda III/IV) are difficult to manage: ultrasound and fine-needle aspiration (FNA) often yield non-definitive results, so many patients undergo diagnostic lobectomy for lesions that prove benign. The combination of AI-enhanced ultrasound and molecular biomarker platforms provides complementary diagnostic capabilities. However, evidence on their combined application remains limited, derives from a small number of early studies, and has not yet been validated in large or prospective cohorts.

**Methods:**

A systematic search of PubMed (2012–2025) identified studies evaluating the diagnostic performance of conventional ultrasound + FNA, AI-assisted ultrasound, molecular biomarker platforms (Afirma, ThyroSeq, ThyGeNEXT, ThyraMIR), and integrated multimodal approaches combining AI-enhanced ultrasound radiomics with molecular biomarker testing in indeterminate thyroid nodules. The search was limited to English-language publications. After applying inclusion and exclusion criteria, 49 studies were included. Extracted outcomes included sensitivity, specificity, negative predictive value (NPV), and positive predictive value (PPV). Study quality was appraised using QUADAS-2, and model reporting transparency was benchmarked against TRIPOD and STARD-AI guidelines. Because of methodological heterogeneity, no meta-analysis or statistical pooling was performed; summary values are unweighted descriptive means presented with observed ranges.

**Results:**

Conventional ultrasound combined with FNA cytology demonstrated high sensitivity (71–99.6%) but variable specificity (14.1–92%), reflecting persistent diagnostic uncertainty in Bethesda III/IV nodules. AI-enhanced ultrasound tended to improve specificity and reduce false-positive findings compared with conventional methods, though performance varied considerably across studies. Molecular biomarker platforms showed generally high diagnostic performance, with a descriptive (unweighted) mean sensitivity of 86.8%, specificity 78.5%, and NPV 91.8%, enabling many patients to avoid diagnostic lobectomy. Only a few studies have evaluated integrated AI + biomarker models; these preliminary results suggest potential additive value but require further prospective validation before conclusions can be drawn.

**Conclusions:**

Molecular biomarker testing and AI-assisted ultrasound each show promise for enhancing diagnostic accuracy in indeterminate thyroid nodules, and early evidence suggests that integrating these modalities could further refine malignancy-risk assessment. However, the current evidence is preliminary and heterogeneous, and the benefit of combined approaches has not yet been confirmed. Large, multicenter prospective trials with standardized protocols and transparent reporting are needed to confirm these findings and to assess real-world cost-effectiveness before routine clinical adoption.

## Introduction

### Clinical problem: indeterminate thyroid nodules (Bethesda III/IV)

Thyroid nodules categorized as Bethesda III or IV present one of the most difficult challenges in thyroid diagnostics. These nodules carry a variable risk of malignancy but often lack clear cytologic or imaging features to confirm a diagnosis. As a result, many patients undergo diagnostic lobectomy, even though a substantial proportion of these nodules prove benign on final pathology (1, 2). Beyond the unnecessary procedures themselves, this approach carries significant burdens, including surgical risks, lifelong thyroid hormone dependence, increased healthcare costs, and patient anxiety (3).

### Limitations of conventional ultrasound and cytology (FNA)

Ultrasound and fine-needle aspiration (FNA) remain the primary tools for evaluating thyroid nodules, but both have notable limitations in Bethesda III/IV cases. Although the Thyroid Imaging Reporting and Data System (TIRADS) provides structured risk stratification, its accuracy varies widely between institutions and interpreters, often leading to false positives and avoidable procedures (4). FNA also has limited predictive accuracy for follicular-patterned lesions and frequently produces nondiagnostic results, further complicating decision-making (5).

### Current role of molecular biomarker testing (Afirma, ThyroSeq, ThyGeNEXT, ThyraMIR)

Molecular biomarker testing has emerged as an important adjunct in managing indeterminate thyroid nodules. Platforms such as Afirma GSC and ThyroSeq v3 consistently demonstrate high negative predictive values, reducing the need for diagnostic surgery in many patients (6, 7). Multiplex assays that incorporate mRNA, miRNA, and DNA analysis have further refined diagnostic accuracy, supporting a more personalized approach to management (8, 9). Nonetheless, test performance can vary by nodule subtype and across healthcare settings, limiting their reliability as stand-alone tools.

### Advances in AI-enhanced, radiomics-based ultrasound tools

Artificial intelligence (AI) and radiomics have advanced ultrasound imaging by extracting quantitative features not detectable by the human eye. AI-assisted TIRADS classifiers and deep-learning models have consistently outperformed conventional ultrasound in diagnostic accuracy in several studies, particularly for indeterminate nodules (10–12). In some studies, AI-enhanced ultrasound has matched or exceeded FNA performance in malignancy prediction, while reducing interobserver variability and providing more objective risk assessment (13, 14). Clinical trials suggest that AI decision-support systems may improve both sensitivity and specificity in thyroid cancer detection (15).

### Rationale for integrating multimodal diagnostics

AI-driven imaging and molecular biomarker testing offer complementary strengths. Radiomics-based models may improve imaging-based risk stratification, while molecular assays enhance biological characterization of nodules (16). Early integrated models combining these modalities have demonstrated potential for reductions in false positives and improved preoperative decision-making in preliminary studies (16, 17). However, these findings derive from a small number of early, retrospective reports and require prospective validation before clinical recommendations can be made.

The integrated AI + biomarker pathway illustrated in **Figure 1** seeks to overcome the diagnostic limitations of the conventional approach by introducing two high-performance triage points: AI-enhanced ultrasound for initial risk stratification, followed by molecular biomarker testing for indeterminate cases. While AI-driven imaging and molecular platforms have individually demonstrated performance advantages compared with conventional methods in several studies—particularly in reducing false positives and unnecessary surgeries—evidence on their combined application remains limited and early. A formal review is therefore needed to synthesize the existing evidence on these two advanced modalities and to assess the effect of their integrated use on diagnostic accuracy and clinical outcomes for Bethesda III/IV nodules.

**Figure 1:**
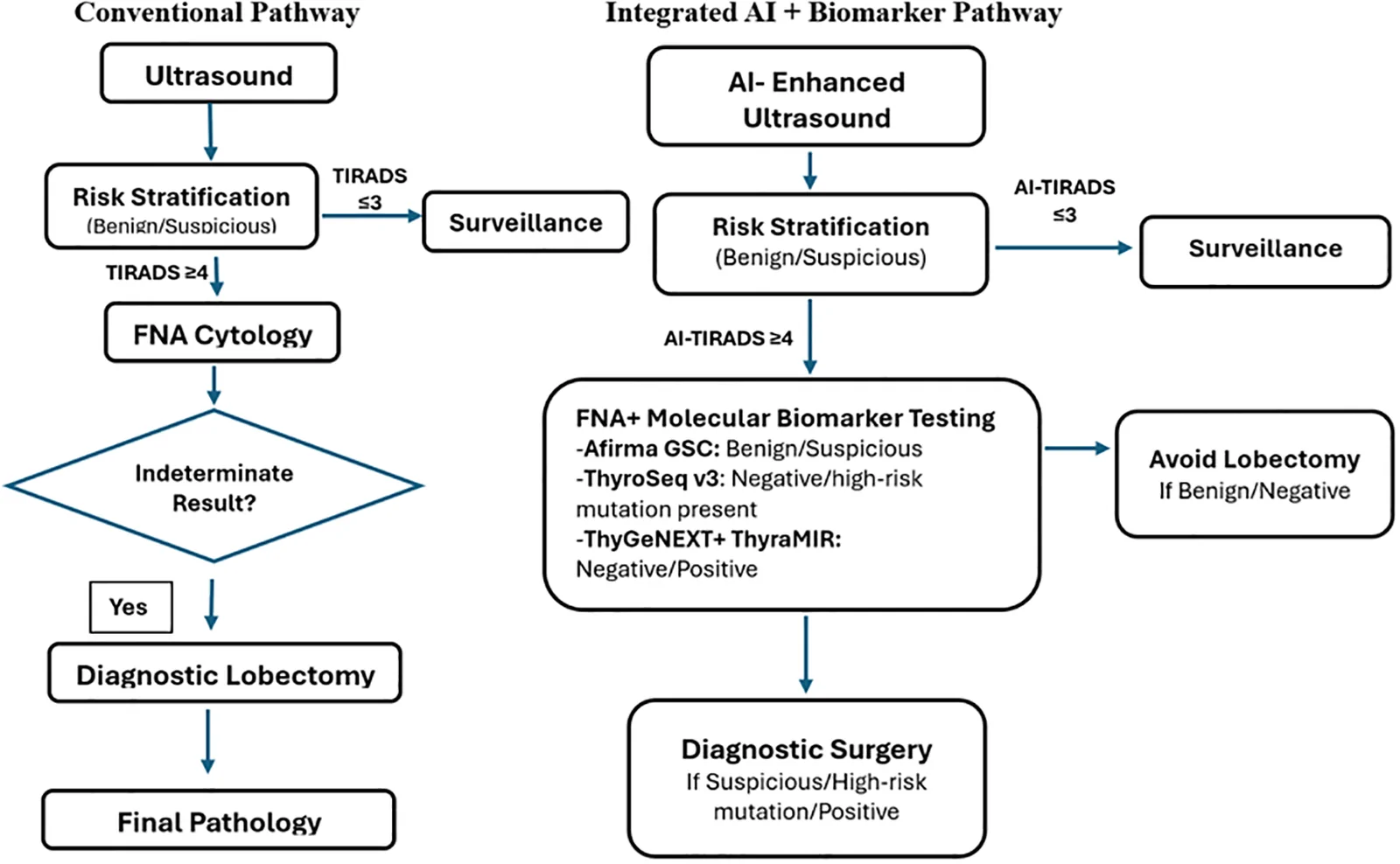
Comparison of conventional and integrated diagnostic workflows for indeterminate thyroid nodules (Bethesda III/IV). The conventional pathway relies on ultrasound and FNA cytology, often leading to indeterminate results and diagnostic lobectomy. The integrated pathway combines AI-enhanced ultrasound with molecular biomarker testing to refine risk stratification and guide management decisions prior to surgery.

### Objective of this review

The objective of this review is to evaluate the evidence supporting the combined use of AI-enhanced ultrasound and molecular biomarker testing in Bethesda III/IV thyroid nodules. Specifically, we examine whether this multimodal approach may improve diagnostic accuracy and reduce unnecessary surgical intervention compared with conventional ultrasound and cytology alone.

## Methods

This review was not prospectively registered on PROSPERO or another systematic-review registry; this is addressed as a limitation below.

### Search strategy and study selection

A systematic literature search was conducted using PubMed to identify studies evaluating diagnostic performance in indeterminate thyroid nodules (Bethesda III/IV). The search was conducted from January 1, 2012 to December 31, 2025, restricted to English-language publications. Search terms included combinations of “indeterminate thyroid nodule,” “Bethesda III/IV,” “radiomics,” “AI ultrasound,” “Afirma,” “ThyroSeq,” “ThyGeNEXT,” and “ThyraMIR.” The complete Boolean search string, field tags, filters, and date and language limits applied in PubMed are provided in **Supplementary Material (Search Strategy S1)**. The search was limited to PubMed; consequently, relevant studies indexed in Embase, Scopus, Web of Science, IEEE Xplore, and other databases—particularly AI and radiomics work published in engineering and informatics journals—may not be fully captured. Records identified through the database and manual searches yielded 168 records.

Date range: January 1, 2012 to December 31, 2025. Language restriction: English only.

Eligible studies reported quantitative diagnostic metrics—sensitivity, specificity, PPV, or NPV—for conventional ultrasound, FNA, AI-enhanced ultrasound, molecular biomarker testing, or combined multimodal approaches. Excluded studies were reviews, case reports, editorials, or studies lacking quantitative diagnostic data.

### Eligibility criteria (PICOS)

Population: patients with cytologically indeterminate thyroid nodules (Bethesda III/IV). Index tests: conventional ultrasound/TIRADS, FNA cytology, AI-enhanced ultrasound (radiomics or AI-TIRADS), molecular biomarker platforms, and integrated AI + biomarker models. Comparator: conventional ultrasound and/or FNA cytology, or final histopathology where used as reference. Outcomes: sensitivity, specificity, PPV, NPV. Study designs: prospective and retrospective diagnostic-accuracy and comparative studies.

Eligible designs included prospective and retrospective diagnostic-accuracy, validation, cohort, and comparative studies. Review articles, editorials, letters, case reports or case series without quantitative diagnostic performance, and studies without extractable diagnostic metrics were excluded. Adult and pediatric cohorts were eligible if they met the nodule/cytology criteria; however, the included studies were adult or predominantly adult cohorts unless otherwise specified. Bethesda III and IV data were analyzed together when studies reported combined indeterminate-cytology performance; when available, Bethesda III and IV subgroup data were extracted separately. For studies enrolling broader Bethesda categories or unselected thyroid nodules, only Bethesda III/IV subgroup data were extracted when isolable. Studies were excluded from the quantitative summary when indeterminate-nodule data could not be isolated. Studies with mixed or non-indeterminate cohorts were retained only for narrative context unless Bethesda III/IV-specific diagnostic metrics were extractable.

Titles and abstracts were screened independently by four reviewers. Full-text eligibility was assessed independently by two reviewers, and disagreements were resolved by consensus with senior-author adjudication when needed. Data extraction was performed using a standardized extraction form and verified by a second reviewer. Formal inter-rater agreement statistics, including Cohen’s or Fleiss’ kappa, were not calculated.

### Data extraction and synthesis

Full-text articles of relevant studies were reviewed for eligibility. A data-extraction form was used to collect key variables: study ID (first author, year), population characteristics (sample size, demographics), modality tested (e.g., conventional US, AI-enhanced US), and the primary outcome metrics (sensitivity, specificity, PPV, NPV).

When a study reported diagnostic performance at multiple thresholds, we extracted the value corresponding to the threshold designated by the study authors as primary or clinically recommended. When no primary threshold was designated, we recorded the full reported range rather than selecting a single best-performing value. No threshold was selected solely because it maximized diagnostic performance.

Because of methodological heterogeneity, no meta-analysis or statistical pooling was performed. Where summary values are reported, they represent unweighted descriptive means of study-level estimates and are presented with observed ranges; they should not be interpreted as pooled or weighted estimates. Studies were grouped by modality: (1) conventional ultrasound ± FNA cytology; (2) AI-enhanced ultrasound (radiomics or AI-based TIRADS); (3) molecular biomarker platforms (Afirma GSC, ThyroSeq v3, etc.); and (4) integrated AI + biomarker models.

### Quality assessment

Quality and transparency were evaluated using three frameworks: QUADAS-2 for diagnostic bias, TRIPOD for predictive-model reporting, and STARD-AI for AI-specific transparency. QUADAS-2 scores were tabulated for risk of bias across four domains: patient selection, index test, reference standard, and flow/timing. TRIPOD and STARD-AI adherence were assessed qualitatively based on completeness of model reporting and algorithm description.

## Results

### Study selection and characteristics

The systematic search yielded 168 records, of which 145 remained after duplicates were removed. After screening and applying inclusion/exclusion criteria, 49 studies were included in the final analysis. A PRISMA flow diagram of the selection process is provided in **Figure 2**.

**Figure 2:**
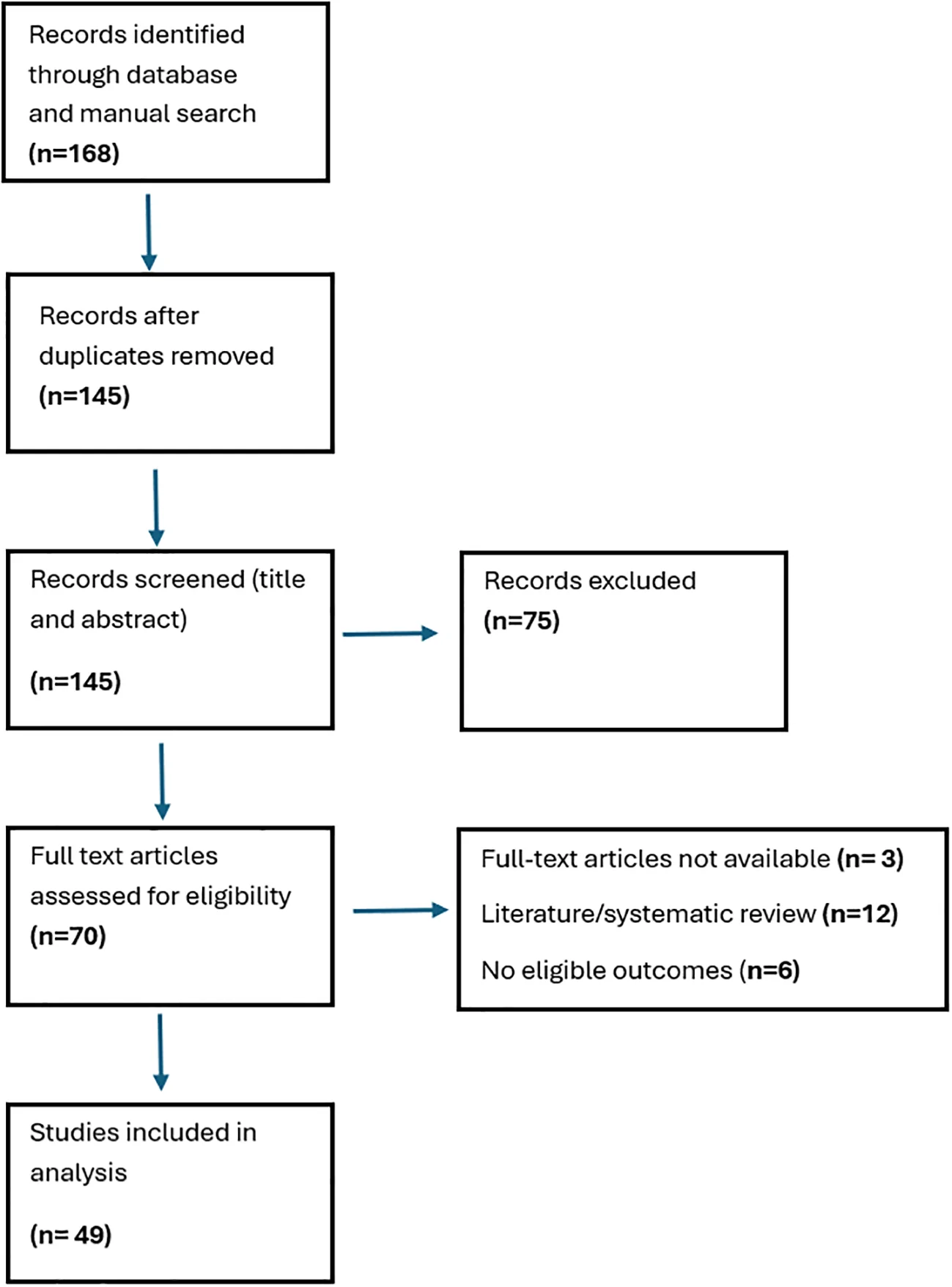
PRISMA flow diagram of study selection process.

### Quality and transparency of evidence

As summarized in **Table 1**, the QUADAS-2 assessment identified verification bias as the most frequent issue (flow-and-timing domain). This high risk of bias was due to partial verification, as only patients who underwent surgery received confirmation with the reference standard (histopathology). This design systematically excludes nodules managed non-surgically (presumably benign), leading to an overestimation of malignancy prevalence and potentially inflated sensitivity estimates. Few studies reported blinding between imaging and cytologic interpretation. The risk of bias in the patient-selection and reference-standard domains was generally low.

**Table 1:** Risk-of-bias assessment using QUADAS-2.

Domain	Overall rating	Key source of bias	Risk (high/low)
Patient selection	Low	Consecutive or well-defined cohorts	9 High/38 Low
Index test	Unclear	Blinding seldom reported	0 High/3 Low
Reference standard	Low–moderate	Histopathology usually used	17 High/32 Low
Flow & timing	High	Verification bias – surgical cases only	31 High/18 Low

TRIPOD adherence was partial, particularly for model calibration and validation. No AI studies fully adhered to STARD-AI requirements, with algorithm training data and threshold reporting inconsistently described. TRIPOD was applied only to model-based studies (**Table 2**).

**Table 2:** Reporting transparency (TRIPOD/STARD-AI).

Guideline component	Comment	Score (satisfied/total)
TRIPOD – Model development & predictors	Validation/calibration often incomplete	12/27
TRIPOD – Handling of missing data	Rarely described	0/27
STARD-AI – Algorithm training/validation	Minimal reporting of dataset splits	12/27
STARD-AI – Decision thresholds & explainability	Not reported	0/27

The incompleteness of STARD-AI and TRIPOD reporting is not a peripheral observation but a direct constraint on interpretation. Where dataset splits, training data, validation strategy, decision thresholds, calibration, and missing-data handling are inconsistently or not reported, the reported diagnostic metrics cannot be assumed to reflect performance in independent populations, and the risk of optimistic or overfit estimates cannot be excluded. The AI performance figures in this review should therefore be read as upper-bound, hypothesis-generating estimates rather than validated accuracy.

As shown in **Table 3**, conventional ultrasound combined with FNA cytology demonstrates high sensitivity but variable specificity, which contributes to false-positive results and unnecessary diagnostic surgeries. AI-enhanced ultrasound models generally show comparable sensitivity with greater specificity and reproducibility across observers, though findings varied substantially between studies. Molecular biomarker assays such as Afirma GSC and ThyroSeq v3 consistently exhibit high negative predictive values, often exceeding 90%, indicating a strong ability to rule out malignancy. Preliminary evidence on integrated AI + biomarker approaches suggests potential improvements in overall accuracy; however, available data remain limited to small, single-center studies, and the differing malignancy prevalence and case mix across modality groups preclude direct numerical comparison.

**Table 3:** Diagnostic performance across modalities for indeterminate thyroid nodules (Bethesda III/IV).

Modality	Representative studies N	Sensitivity % range (mean)	Specificity % range (mean)	PPV % range (mean)	NPV % range (mean)	Malignancy prevalence
Conventional US + FNA cytology	3, 4, 18, 19	71–99.6 (87.5)	14.1–92 (58.3)	25.3–91 (58.7)	82.6–96.6 (89.6)	42.6
AI-enhanced US (radiomics, AI-TIRADS)	10, 14, 19, 20	45–100 (82.5)	45.5–93.4 (77.2)	43.2–95.2 (76.3)	54.5–96.1 (78.9)	37.9
Molecular biomarker platforms (Afirma GSC, ThyroSeq v3, ThyGeNEXT/ThyraMIR)	6, 7, 21–23	53.8–100 (86.8)	39–100 (78.5)	37.4–97.8 (73.0)	67.4–100 (91.8)	32.5
Integrated AI + biomarker	16, 24	73.1 (Zhong); 100 (Wang)	79.6 (Zhong); 84.6 (Wang)	63.3 (Zhong); 97.3 (Wang)	NR (Zhong); 100 (Wang)	84.7

US, ultrasound; FNA, fine-needle aspiration; AI, artificial intelligence; NPV, negative predictive value; PPV, positive predictive value; NR , not reported.

Values are reported as observed range, with the unweighted descriptive mean in parentheses; these are not pooled or weighted estimates. PPV and NPV are prevalence-dependent and are not directly comparable across modality groups with differing malignancy prevalence and case mix. NPV for Zhong 2024 was not reported (NR) in the original publication.

Preliminary integrated-model studies suggest that multimodal AI-radiomics and molecular/clinical approaches may improve risk stratification and could reduce unnecessary diagnostic surgery, but available studies are insufficient to establish a reproducible magnitude of benefit. This outcome requires prospective validation in cohorts designed to measure surgical decision-making and downstream patient outcomes.

### Diagnostic performance of conventional ultrasound and FNA cytology

This subsection separates three distinct diagnostic functions that are often conflated: imaging-based risk stratification, cytologic diagnosis, and the combined pathway confirmed by surgical histopathology.

#### Imaging-based risk stratification (ultrasound/TIRADS and modified scoring)

Conventional ultrasound (US) serves as the standard pre-FNA imaging modality for risk stratification, yet its diagnostic performance varies widely depending on operator expertise, scoring system, and nodule composition. He et al. (21) evaluated indeterminate nodules using TI-RADS and a modified scoring system. TI-RADS alone produced near-perfect sensitivity (99.6%) but very low specificity (14.1%, PPV 59.9%), whereas a new scoring model improved specificity to 90.4% with 78.4% sensitivity (PPV 95.7%). A combined TI-RADS and scoring model achieved balanced performance (97.6% sensitivity, 48.5% specificity, PPV 70.9%). Talmor et al. (2022) found that individual sonographic features had modest standalone utility: microcalcifications offered 47.4% sensitivity and 74.4% specificity, irregular margins only 14.9% sensitivity but 94.4% specificity, and a three-feature combination yielded 98.3% specificity with just 8.8% sensitivity. Sahli et al. (2019) reported that TI-RADS classification alone provided only moderate sensitivity (73%) and poor specificity (36.9%), with PPVs of 25–41%, underscoring the limited discriminatory value of ultrasound in this high-risk subgroup.

#### Cytologic diagnosis (FNA)

Between 15% and 30% of FNA samples yield Bethesda III/IV results, with a malignancy risk of 20–30% (5). As a result, many patients undergo diagnostic lobectomy only to receive benign final pathology, reflecting the limited predictive accuracy of cytology alone in follicular-patterned and indeterminate lesions.

#### Combined US + FNA pathway with histopathologic confirmation

Across five representative studies, combined US + FNA achieved consistently high sensitivity (71–99.6%) and variable specificity (14.1–92%). Liu et al. (19) assessed a larger mixed cohort using both cytology and histopathology as reference standards and found stronger combined performance (96.1% sensitivity, 77.7% specificity), demonstrating that integrating ultrasound interpretation with FNA and histologic confirmation yields greater diagnostic reliability than ultrasound assessment alone. Because histopathologic confirmation was generally available only for surgically resected nodules, these combined-pathway estimates are subject to verification bias (see Discussion).

### Diagnostic performance of molecular biomarker platforms

Molecular biomarker platforms differ substantially in technology, generation, and intended diagnostic role and should not be treated as equivalent. RNA-based gene-expression classifiers (e.g., Afirma GSC) and high-NPV genomic classifiers function principally as rule-out tests, supporting avoidance of diagnostic surgery in molecularly benign nodules. Mutation/fusion panels (e.g., ThyroSeq v3, ThyGeNEXT/ThyraMIR) provide additional rule-in information (25) when high-risk alterations (e.g., BRAF V600E, high-risk variants) are present. Older platforms (e.g., the first-generation Afirma Gene Expression Classifier, GEC) should not be treated as equivalent to current assays, given the improved specificity of later generations (e.g., Afirma GSC).

ThyroSeq v3 has shown consistently high diagnostic accuracy (26). In a cohort of 276 indeterminate FNAs, Sirotnikov et al. (27) reported that 42% of nodules harbored genetic alterations; high-risk mutations conferred a 100% risk of malignancy, while RAS-like variants carried a 77.5% risk when including NIFTP. Overall, ThyroSeq v3 achieved a PPV of 78.2% and an NPV of 75.9% in Bethesda III/IV nodules. Han et al. (21) randomized 346 indeterminate nodules to Afirma GSC (RNA-based) or ThyroSeq v3 (DNA/RNA-based) testing and observed no significant difference in sensitivity (100% vs 96.9%) or specificity (79.6% vs 84.8%); nearly half of patients (48.6%) with negative molecular results avoided diagnostic surgery.

Afirma GSC and Xpression Atlas also demonstrate robust performance (28, 29). Hu et al. (29) found that approximately half of Bethesda III–VI nodules classified as “suspicious” by GSC harbored actionable molecular alterations. Kim et al. (6) tracked Bethesda III/IV nodules with benign Afirma GSC or ThyroSeq v3 results for a median of 34 months and identified only one malignancy (0.6%) during surveillance, supporting the high NPV of modern molecular testing. Large-scale and quality-assurance studies (30–32) suggest that integrating cytomorphology with mutation profiling reduces diagnostic variability. Historical data confirm clinical impact: following introduction of the Afirma GEC, Yang et al. (8) observed that 42% of indeterminate nodules were reclassified as benign, and Sacks et al. (33) reported that excision rates for AUS/FLUS lesions dropped from 63% to 35%, with the malignancy rate in operated nodules rising from 35% to 47%, reflecting improved surgical selection. Steward et al. (23) showed that most benign-classified nodules were safely monitored without surgery.

Collectively, these studies indicate descriptive mean sensitivities around 87%, specificities around 79%, and NPVs exceeding 90% for molecular platforms in Bethesda III/IV nodules, supporting their value primarily as rule-out tools (22, 34).

### Diagnostic performance of AI-enhanced ultrasound

Across the included AI-based studies, sample sizes ranged from small single-center cohorts of fewer than 30 patients to multi-institutional datasets of more than 1,600 patients (35–41). Performance metrics varied widely with study design, dataset size, and malignancy prevalence. Most AI models demonstrated high sensitivity, with several studies reporting values above 90% (11, 14, 19). For example, Liu et al. (19) reported sensitivity of 96.1% and specificity of 77.7% in 555 nodules, while Zhou et al. (14) showed sensitivity of 96.3% with specificity of 89.1% in a mixed retrospective–prospective dataset. By contrast, early or smaller studies reported much lower sensitivity (as low as 45–50%), suggesting that AI model maturity and sample size are strong predictors of performance. Specificity ranged from 45.5% to 94%, likely due to differences in model training and validation.

PPVs were inconsistent and dependent on disease prevalence: many small-sample studies reported low PPVs (e.g., 43.2%), while larger, higher-prevalence cohorts reported values exceeding 90%. NPVs were more stable (80–96%), suggesting AI may be useful for ruling out malignancy; an outlier NPV of 54.5% likely reflected a small sample (27 patients). Several studies compared AI classifiers against radiologists. Liang et al. (42) demonstrated a CAD system with higher sensitivity than radiologists (64% vs 61% for experts and 57% for novices) while maintaining slightly better specificity. Du (10) showed that a deep-learning radiomics nomogram improved radiologist performance at all levels, increasing AUCs for junior clinicians from 0.71–0.75 to as high as 0.92 with AI assistance. Models combining radiomics with clinical features generally outperformed single-input models: D’Andréa et al. (43) reported strong NPVs (~80%) using multimodal inputs, and Cai et al. (20) validated a random-forest model across three cohorts with accuracy of 90–97% and AUCs up to 0.994, although external generalizability is limited. Across studies, limitations included small sample sizes, retrospective single-center design, a bias toward surgically treated or high-malignancy cohorts, and inter-institutional variability in ultrasound equipment and acquisition protocols. Few studies reported cost-effectiveness.

### Integrated approaches (AI + biomarkers)

Only two included studies directly evaluated the integration of AI-based ultrasound radiomics with molecular biomarker testing (**Figure 3**); both were retrospective and single- or bi-center, with limited or population-specific external validation. Their results should therefore be regarded as preliminary and hypothesis-generating.

**Figure 3:**
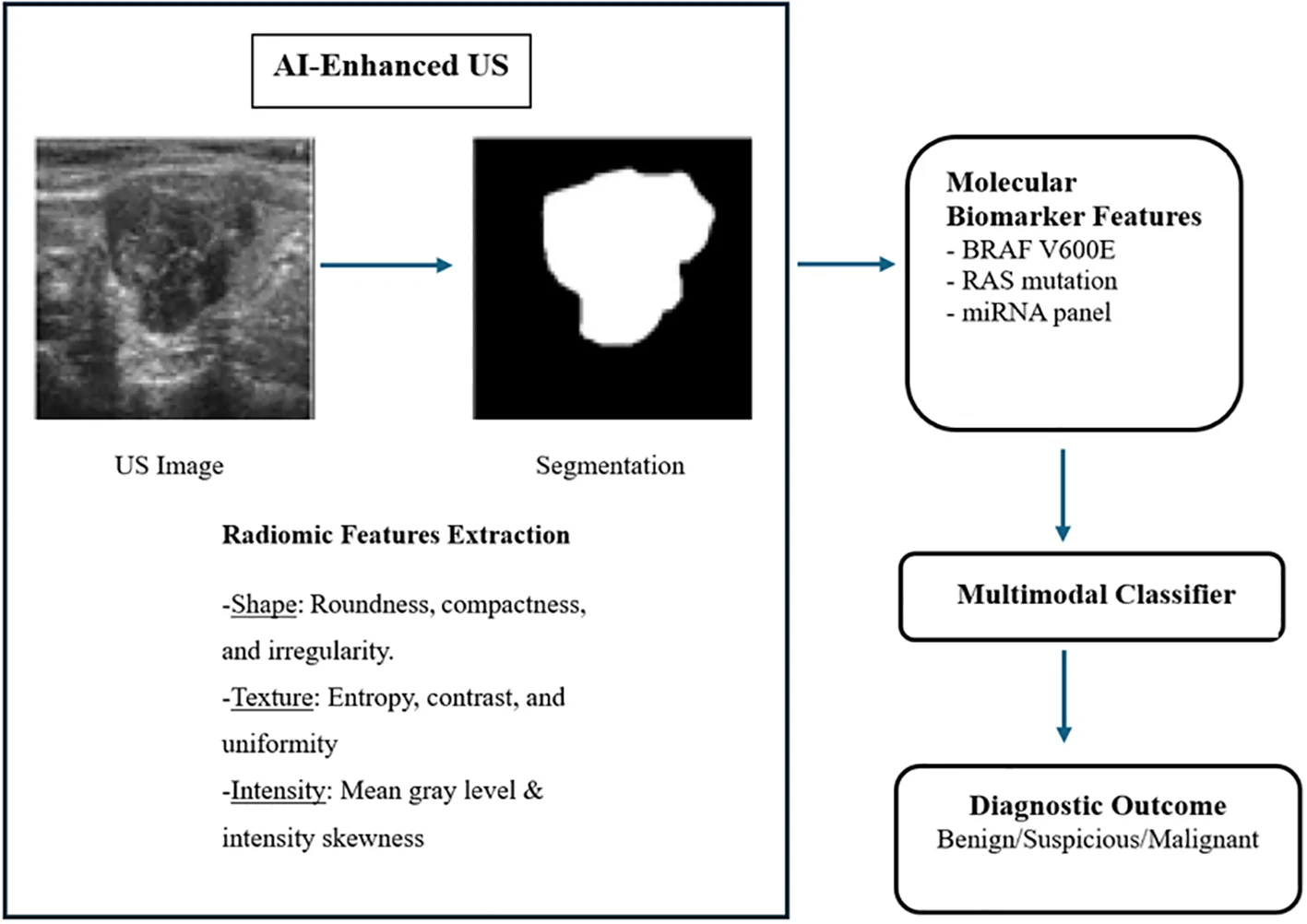
Conceptual fusion model illustrating the integration of radiomic imaging features and molecular biomarker signatures in the diagnostic classification of indeterminate thyroid nodules (Bethesda III/IV). Radiomic features extracted from AI-enhanced ultrasound, such as shape, texture, and echogenicity, are combined with molecular data derived from biomarker assays (Afirma GSC, ThyroSeq v3, ThyGeNEXT, ThyraMIR).

Wang et al. (16) compared radiomics-only, molecular-only, and combined models in indeterminate nodules. The radiomics model achieved good sensitivity (86%) but low specificity (17.6%), while a molecular model incorporating CLDN10, HMGA2, LAMB3, and BRAF V600E reached 85.9% accuracy. When merged into the integrated C-thyroid model, diagnostic accuracy improved to 96.7% overall accuracy, 100% sensitivity, and 93.8% specificity in the training set, and 97.6% accuracy with 100% sensitivity in validation; the integrated model correctly identified 9 of 10 indeterminate nodules. Zhong et al. (24) reported, in a bi-center cohort of Bethesda III nodules, that an integrated clinical-radiomics nomogram incorporating BRAF V600E status yielded the highest AUC (0.824) and accuracy (0.775) across internal and external test sets, outperforming clinical-only or radiomics-only models; BRAF V600E was a significant independent predictor (P = 0.02). NPV was not reported by Zhong et al. Together, these two studies suggest potential additive value of combining AI-enhanced ultrasound with molecular analysis, but this has not been confirmed in large or prospective cohorts.

## Discussion

### Principal findings

This review indicates that integrating AI-enhanced ultrasound with molecular biomarker platforms has the potential to improve diagnostic accuracy in indeterminate thyroid nodules compared with conventional ultrasound and cytology alone. AI-based radiomics and TIRADS classifiers generally tended to increase specificity, reducing false-positive rates and, in turn, the number of unnecessary FNAs in several studies (10, 12, 13). Conventional ultrasound and FNA cytology serve as the baseline pathway—with a descriptive mean sensitivity of 84.5% and mean specificity of 71.1% across the studies analyzed—but their lower specificity contributes to unnecessary diagnostic surgeries. Molecular biomarker studies demonstrated the highest descriptive mean sensitivity and NPV (86.8% and 91.8%, respectively). Although data on integrated AI + biomarker models remain limited, the strong rule-out capability of molecular assays and the improved specificity of AI-enhanced ultrasound suggest that a combined modality could reduce the number of required FNAs and diagnostic lobectomies. These inferences remain preliminary, given the small number of integrated studies, the absence of prospective validation, and the heterogeneity of study designs.

### Verification bias and its differential effect across modalities

Verification (partial-verification) bias was the most frequent source of bias across included studies, because histopathologic confirmation was generally available only for surgically resected nodules, while clinically followed nodules—presumably enriched for benign disease—were excluded. The expected effects differ by modality and metric. Inflated malignancy prevalence tends to raise PPV and lower NPV relative to a true unselected indeterminate population. For conventional ultrasound/FNA, this may overstate apparent sensitivity. For molecular platforms, whose principal claim is high NPV as rule-out tests, a surgical-only spectrum may understate real-world NPV (because truly benign, non-operated nodules are absent), while inflating apparent malignancy detection. For AI and integrated models trained and tested on surgical cohorts, performance is likely optimistic and may not generalize to the lower-prevalence population actually triaged in practice. These prevalence-dependent distortions caution against direct cross-modality comparison.

### Clinical implications: unnecessary surgery, patient outcomes, and cost-effectiveness

Avoiding diagnostic lobectomy directly addresses one of the greatest shortcomings of current practice, where a substantial proportion of Bethesda III/IV nodules prove benign after surgery (1, 2). Reducing avoidable surgeries spares patients surgical complications, preserves thyroid function, and minimizes psychological and financial burdens (3). Multimodal diagnostics also support a more individualized approach to care: combining imaging-based risk stratification with molecular signatures may help clinicians tailor management while balancing overtreatment against the risk of missing malignancy. Early analyses suggest that combining AI-enhanced ultrasound with molecular testing could potentially improve cost-effectiveness by decreasing unnecessary biopsies and surgeries in some settings (16, 17), though the magnitude of any savings will depend on local healthcare-system structure, test availability, and patient demographics, and this remains unproven.

Preliminary integrated-model studies suggest that multimodal AI-radiomics and molecular/clinical approaches may improve risk stratification and could reduce unnecessary diagnostic surgery, but the available studies are insufficient to establish a reliable estimate of the magnitude of benefit. This outcome requires prospective validation in cohorts designed to measure surgical decision-making and downstream patient outcomes.

### Comparison with AI integration in other cancer diagnostics

AI integration is more mature in breast and lung cancer. In breast ultrasound, an AI-CADx system (BU-CAD) achieved 91.1% sensitivity, 92.4% specificity, and an AUC of 0.948, comparable to experienced radiologists and faster than less-experienced readers (44). In digital breast tomosynthesis, deep-learning models reached AUCs around 0.93, with radiologist AUC improving from 0.90 to 0.92 with AI support. Large-scale screening AI has shown AUCs up to 0.96, with combined human–AI sensitivities around 88.6% and specificities around 93.0% (45, 46). In lung cancer, a meta-analysis of AI-aided CT screening reported pooled sensitivity of 94.6% and specificity of 93.6% (47), and AI systems detected nodules missed by radiologists while being significantly faster (48). These examples illustrate the potential for analogous benefits in thyroid diagnostics with further development and evidence.

### Barriers to implementation in routine practice

Key barriers include reliance on small, homogeneous, or single-center datasets that limit generalizability (49); limited transparency and explainability, which undermines clinician trust (50); a scarcity of prospective, multicenter validation and of evidence on patient-relevant outcomes (50); poor workflow and PACS/EHR integration (51); regulatory, legal, and ethical uncertainties around software as a medical device, data privacy, liability, and training-data bias (52); upfront cost and resource constraints, particularly for smaller institutions (53); and gaps in education, training, and stakeholder acceptance.

### Future research priorities

Priorities include constructing large, multicenter, heterogeneous datasets spanning different ultrasound machines, demographics, and routine practice; fusing multimodal data (imaging plus clinical and molecular information) rather than relying on imaging alone (54); improving explainability and interpretability (55); conducting prospective clinical-utility and validation studies under real-world conditions (56); standardizing imaging protocols, feature annotation, and ground-truth definitions (57); developing robust post-deployment performance monitoring to detect drift and bias (58); and performing cost-effectiveness, workflow-integration, and clinician-acceptability studies (59).

### Limitations of this review

This review has several limitations. First, it was not prospectively registered on PROSPERO or another registry. Because the eligibility criteria, outcomes, and analysis plan were therefore not publicly pre-specified, the review is more vulnerable to *post-hoc* selection of studies and outcomes—a concern heightened by the heterogeneity of the diagnostic modalities compared. Second, our search was restricted to PubMed. Relevant studies indexed in Embase, Scopus, Web of Science, the Cochrane Library, IEEE Xplore, and clinical trial registries—including AI and radiomics work published in engineering, imaging, and informatics journals—may not be fully captured, which limits comprehensiveness and is a recognized source of selection bias.

Third, many included studies had very small sample sizes (some fewer than 50 patients, including cohorts of 45, 33, and 10), limiting generalizability. Heterogeneous inclusion/exclusion criteria and outcome reporting hindered direct comparison and reproducibility, and some studies did not report radiomics- or molecular-model performance separately. Fourth, BRAF V600E prevalence differs markedly between populations (approximately 45% among patients with papillary thyroid carcinoma in the United States versus 58–63.7% in some East Asian cohorts), and many included studies were conducted in East Asian populations with higher malignancy prevalence; broader, more diverse validation is needed. Finally, no meta-analysis was performed because of methodological heterogeneity, so summary values are descriptive only.

### Standardization: requirements for clinical adoption

A major barrier to adopting AI- and biomarker-based diagnostic tools for indeterminate thyroid nodules is the lack of standardization. Imaging protocols, AI training methods, and biomarker assays varied widely, producing inconsistent and hard-to-reproduce results. Standardized pipelines for AI training, radiomics feature selection, and diagnostic reporting—together with consistent inclusion/exclusion criteria—are essential to produce reliable, clinically relevant, and reproducible data. These limitations underscore the need for large, multicenter, prospective trials with standardized protocols to validate AI–biomarker integration in diverse patient populations.

## Conclusion

This systematic review evaluated the diagnostic performance of conventional ultrasound, FNA cytology, AI-enhanced ultrasound, molecular biomarker platforms, and their integration in the management of indeterminate thyroid nodules (Bethesda III/IV). Conventional ultrasound with FNA provides important baseline information but is limited by substantial diagnostic uncertainty: sensitivity is often high, but poor specificity drives unnecessary diagnostic lobectomies, with 20–30% malignancy rates meaning that most operated patients have benign disease (2, 5). AI-enhanced ultrasound generally showed improved specificity and reduced false positives in several cohorts, and in some studies matched or exceeded FNA while reducing interobserver variability. Molecular biomarker platforms, including Afirma GSC, ThyroSeq v3, and combined microRNA/mutation panels, achieved high sensitivities and NPVs in the mid-90s in several studies, functioning as reliable rule-out tools; however, PPV remains variable and interpretation must consider nodule subtype, malignancy prevalence, and institutional context.

These technologies are promising but not yet ready for routine clinical adoption. The current evidence is constrained by substantial between-study heterogeneity, incomplete AI reporting (STARD-AI/TRIPOD), pervasive verification bias, limited prospective and external validation, and a near-absence of standardized imaging and molecular protocols. Only a small number of integrated studies exist, all retrospective and single- or bi-center. Before AI-enhanced ultrasound and integrated AI–molecular pathways can be recommended for routine indeterminate-nodule management, the field requires large prospective multicenter validation, standardized imaging and molecular protocols, transparent and complete AI reporting, formal cost-effectiveness analysis, and real-world workflow studies. This review should be read as a synthesis of promising but preliminary evidence rather than as support for current clinical implementation.

## Data Availability

This systematic review extracted and summarized study-level diagnostic data from published literature. No original datasets were generated or analyzed for this study; all data were drawn from previously published sources.
