# Supplementary material for: Integration of AI-driven ultrasound and molecular testing to improve diagnosis of indeterminate thyroid nodules
Source: Front Endocrinol (Lausanne). 2026 Sep 25;17:1764495. doi: 10.3389/fendo.2026.1764495 (PMC13649419; doi:10.3389/fendo.2026.1764495)
Supplement: Supplementary file 1 [file Table1.docx]

***Comparison with AI integration in other cancer diagnostics***

AI integration in other cancer diagnoses includes notable improvements in both lung and breast cancer. In the diagnosis of breast cancer, AI has been largely advanced, particularly in ultrasound and mammography-based diagnosis. For example, in breast ultrasound, an AI-CADx system (BU‑CAD™) achieved 91.1% sensitivity, 92.4% specificity, and an AUC of 0.948 which reads like veteran radiologists and more accurately than less veteran readers. It also reduced interpretation time considerably (44). Similarly, in digital breast tomosynthesis (DBT), deep learning models as a standalone device have also achieved AUCs of around 0.93, with radiologists showing improvement in diagnostic performance with AI support. For instance, one study showed radiologist AUC increase from 0.90 to 0.92, with specificity increase and reading time decrease. This is a relevant study because it directly compares the AI-based breast ultrasound diagnostic systems to radiologists and evaluates the incremental benefit of AI assistance, aligning with the focus on the integration of AI, rather than standalone performance. Tsuyuzaki et al. first demonstrate that AI can achieve accuracy comparable to that of radiologists and then improves diagnostic performance and efficiency as a support tool, reinforcing evidence that AI meaningfully enhances the workflows of breast cancer detection, similar to advances seen in other cancers.

For breast cancer screening on a large scale, commercially available AI algorithms have shown AUCs as great as 0.96 in retrospective studies. When combined with radiologists, the systems have shown combined sensitivities of around 88.6% and specificities of 93.0%, showing outstanding performance in detecting malignancies without generating too many false positives (45). Some AI software in population screening settings has also shown increased cancer detection without concurrent excessive false-positive results, making diagnostic pathways more effective and precise (46). Taya et al. are relevant to your research because they compare different AI approaches for screening mammography, proving that high AUC values can be obtained even with commercial AI solutions available on the market. This corresponds with your argument that AI has matured enough to be used on a population level with a similar performance to that of professional radiologists. Similarly, the work of Seker et al. is highly pertinent, as it assesses AI within the context of a real-world setting, specifically within a mammography program for breast cancer, and shows how a secondary interpreter such as AI can raise cancer detection rates when implemented within a real-world setting, as opposed to a controlled one. The observation that sensitivity is increased overall with no marked increase in false positives supports your argument that AI-improved screening enhances efficiency.

Diagnosis of lung cancer has also seen significant integration of AI, especially in the use of CT imaging for screening and nodule classification. Meta-analysis of AI-aided lung cancer screening via CT imaging reported pooled sensitivity of 94.6% and specificity of 93.6% (47). For pulmonary nodule detection and classification, AI systems consistently match or outperform humans with greater sensitivity but sometimes reduced specificity relative to radiologists. An experiment noted that AI programs were capable of detecting nodules that radiologists had missed and were significantly faster, especially in high-volume settings (48). These are highly relevant studies because they provide robust evidence that AI integration in lung cancer diagnosis using CT imaging achieves high diagnostic accuracy at scale, supporting the discussion of AI as a reliable screening and detection tool. Thong et al. present a meta-analysis that synthesizes multiple studies with consistently high pooled sensitivity and specificity, enhancing the generalizability and clinical credibility of AI-assisted lung cancer screening. Abadia et al. further reinforces the idea that AI systems are not inferior to traditional radiology readings in detecting lung nodules even among those with complex lung disease, which further points toward real-world applicability. These references put together substantiate the argument that AI enhances diagnostic performance and efficiency in lung cancer and provides a transferable framework for applying similar AI methodologies to other diseases, such as thyroid pathology.

By way of lung and breast cancer diagnosis, AI implementations have been proven to have far more than rudimentary detecting abilities, facilitating workflow, establishing standardized interpretation, and assisting in risk stratification. These examples set the stage for AI potential for other diseases, such as thyroid disease, where the same benefits can be derived with further development and evidence.

***Barriers to Implementation in Routine Practice***

One of the main challenges is data-related issues, specifically small, homogeneous, or single-center datasets that limit generalizability. For AI in thyroid imaging/histopathology, research is more likely to be based on retrospectively acquired data from a single center, unvalidated externally, and over a narrower range of disease presentations. These limitations reduce the extent to which an action will be reliable if taken in a new clinical setting (49). This scoping review is relevant because it systematically identifies barriers relating to data, such as small, homogeneous, single-center datasets, to clinical adoption and generalizability for AI in breast imaging that directly parallels the challenges observed in AI thyroid research. Lokaj et al. present a validated framework for understanding how inadequate data diversity and a lack of external validation undermine the implementation of reliable AI and support the argument about the reduced transferability of AI models to new clinical settings.

Transparency is another harsh bottleneck. Clinicians do not trust AI models whose decision-making rationales are imperceptible. Without explainable AI or mechanisms for explaining model predictions, users cannot easily accept, verify, or audit results, particularly when clinical stakes are high (50). Validation, generalizability, and clinical utility are also key concerns. Many AI models are retrospectively validated on potentially unrepresentative datasets on which deployment is intended. Multicenter, prospective studies are rare. Furthermore, even if technical performance is good, evidence of the impact on patient outcomes like reducing unnecessary biopsies, morbidity, cost is often weak or lacking (50). This narrative review is highly relevant because it synthesizes current applications of AI in thyroidology while critically discussing the very same limitations with transparency, validation, and generalizability. Toro-Tobon et al. notes that black-box AI models undermine clinician trust and adoption in thyroid care, directly supporting the discussion on the need for explainable AI within high-stakes diagnostic settings. Similarly, the review underscores that many thyroid AI systems are based on retrospective, single-center data sets with limited external validation-reinforcing concerns about real-world reliability. This reference strengthens the argument that further evidence is needed prior to safely and effectively integrating AI into thyroid diagnostic pathways by pointing to gaps in prospective, multi-center validation and downstream patient-centered outcomes.

Another challenge is user experience and workflow integration. Even top performing AI tools can flounder when deployed if they are difficult, slow, poorly integrated with existing radiology or clinical systems (PACS, EHR), or disrupt traditional workflows. Ease of use, efficiency, and compatibility are worth a lot but are often under-prioritized (51). This is an important review because, apart from scrutinizing the diagnostic accuracy of AI models concerning thyroid nodules on ultrasound images, the discussion includes other factors that would impact the implementation of AI models in radiology practices. For instance, Wildman-Tobringer et al. emphasizes that for AI models to be useful, they need to be smoothly integrated into current practices for interpreting ultrasound images, which confirms the point that bad workflow integration can hinder the useful application of models with high performance.

Regulatory, legal, and ethical concerns are relevant. These include uncertainty over how AI tools are regulated (notably "software as a medical device"), data privacy issues (HIPAA, GDPR, etc.), liability in the event that outputs of AI lead to errors, bias in training datasets (leading to discrepancies), and intellectual property / ownership issues. These are issues to both developers and clinical institutions (52). This paper is relevant because it outlines the regulatory, legal, and ethical frameworks that control the deployment of clinical AI including software-as-a-medical-device regulation, liability, and data privacy. Chua et al. give a translational perspective on how these issues affect both developers and healthcare institutions and have reinforced the call for governance and bias to be addressed before general clinical adoption.

Cost and resource constraints are also not insignificant. The implementation of AI systems typically involves up-front expenses: buying or leasing the software, adapting infrastructure (computer power, storage, integration), and maintenance. Smaller or limited-resource institutions may be unable to afford them (53). This article is relevant because it addresses real-world implementation barriers of AI in clinical imaging, but with specific regard to the financial and infrastructural costs. Bahl underlines how up-front costs and later maintenance may disproportionately restrict access for smaller or resource-constrained institutions and directly supports the discussion of cost as one of the huge obstacles that must be overcome to provide equitable AI deployment.

Education, training, and stakeholder acceptance form the other obstacle. Clinicians and staff can be unfamiliar with AI tools, lack trust in them, or be concerned about the loss of professional autonomy. In the absence of education, user training, and early stakeholder involvement in design and deployment, adoption is stunted.

***Future Research Priorities***

A priority is to construct large, multicenter, heterogeneous datasets with images from different ultrasound machines, mixed demographic populations, and routine clinical practice. Various reviews point out that most existing AI models for thyroid disease and thyroid nodules generally are trained on data from a few centers or homogeneous populations, which precludes generalizability.

Increased heterogeneity of datasets such as age, ethnicity, subtypes of thyroid disease and image quality will reduce bias and enhance robustness. Another region of high importance is fusion of multimodal data over utilization of images alone from ultrasound. For thyroid illness, there is little proof that fusion of ultrasound characteristics with patient clinical information (demographics, lab results, risk factors) could make more accurate diagnosis, risk stratification, and outcome prediction (including malignancy probability or post-operative prognosis) possible (54). This article states that most of the available AI models for thyroid disease are trained on small, single, or a handful of center datasets, thus underlining the need for large, multi-center, and diverse datasets: Yang et al. also emphasize the importance of multimodal models that fuse ultrasound imaging with other clinical and laboratory information in AI models, thus supporting the argument that a blend of different types of data is necessary for a better diagnosis for thyroid disease.

Increasing explainability and interpretability of AI models is also underscored. Clinicians are also more likely to adopt tools whose decisions or decision characteristics are partially transparent, especially where these guide management decisions (to biopsy or not biopsy a nodule). Tools such as explainable AI feature importance visualization, or models mimicking well-understood risk stratification systems TI‑RADS may help (55). This review is relevant because it emphasizes how interpretable AI in oncology is, and the more its model decisions become increasingly transparent, the more clinicians will adopt them. Huhulea et al. also discuss strategies such as feature importance visualization and AI models aligned with established risk systems that directly support the argument for incorporating explainability into thyroid AI tools to inform clinical decision-making.

Clinical trials and validation studies among prospective future users must test AI tools under conditions of normal usage: not against retrospective image archives, but against real-time decision making in multi-center workflows by operators of different experience. This will provide proof of clinical utility like reduced unnecessary biopsies, cost savings, patient outcomes and whether AI tools are superior to present standard of care (56). This is a relevant article, as it points to the importance of having a prospective, real-world validation of thyroid AI systems in a multi-site, varying operator-experience setting. Burgos et al. state that such validation is necessary in order to assess the clinical utility, such as patient outcomes, reduced unnecessary biopsies, as well as cost-effectiveness.

Standardization of imaging protocols and data annotation is also an area of interest. In order to enhance the reproducibility of AI tools between sites, there must be standardization in imaging acquisition, features being annotated, and ground truth definition of pathology or follow‑up. This helps with external validity and regulatory approval (57). This article is also relevant because it underlines the aspect of standardized imaging protocols and data annotations that are crucial for the reproducibility of AI tools in various sites. Ou et al. mentions that for a consideration of external validity and regulatory approval, acquisition methods need to be consistent, with feature definitions and ground truth labeling. This directly supports the discussion raised on improving reliability and generalizability in AI-assisted diagnostics.

Developing robust processes for model performance monitoring, post-deployment monitoring, and safety is equally crucial. Once deployed, the performance of AI tools over time in real-world practice needs to be tracked to detect drift such as change in image quality or population, surprise errors or bias, and defend against patient harm (58). This is an important article because it shows how necessary it is to monitor the performance of AI models even after they have been adopted. Tiwari et al. discusses how necessary it is to monitor how AI models drift, how they make errors, and how they might be biased, which further supports my point.

Finally, cost-effectiveness, workflow integration, and clinician acceptability studies are required. Even with high accuracy AI tools, they may fail if they are expensive, difficult to integrate with clinical systems like PACS, EHR or unacceptable to users: patients, radiologists, endocrinologists. Studies on how AI tools affect time, cost, patient satisfaction, diagnostic workflow, and health-economic outcomes will tell us which tools are viable in a clinical environment (59). This article is relevant because it underlines how successful translation of AI depends not only on accuracy but also on cost-effectiveness, workflow integration, and clinician and patient acceptability. Zhang et al. mention that studies are needed which would assess time, cost, patient satisfaction, and health-economic outcomes determine how AI tools are practical and sustainable in the real world of clinical settings.
